# Supplementary material for: Neutralizing antibody activity against 21 SARS-CoV-2 variants in older adults vaccinated with BNT162b2
Source: Nat Microbiol. 2022 Jul 14;7(8):1180–8. doi: 10.1038/s41564-022-01163-3 (PMC9352594; doi:10.1038/s41564-022-01163-3)
Supplement: Supplementary file 1 — Supplementary Figs. 1 and 2. [file 41564_2022_1163_MOESM1_ESM.pdf]

nature microbiology

SUPPLEMENTARY INFORMATION

https://doi.org/10.1038/s41564-022-01163-3

In the format provided by the authors and unedited.

OPEN

Neutralizing antibody activity against 21 SARS-CoV-2 variants in older adults vaccinated with BNT162b2

In the format provided by the authors and unedited

A. Dilution series: 1:40 – 1:2560

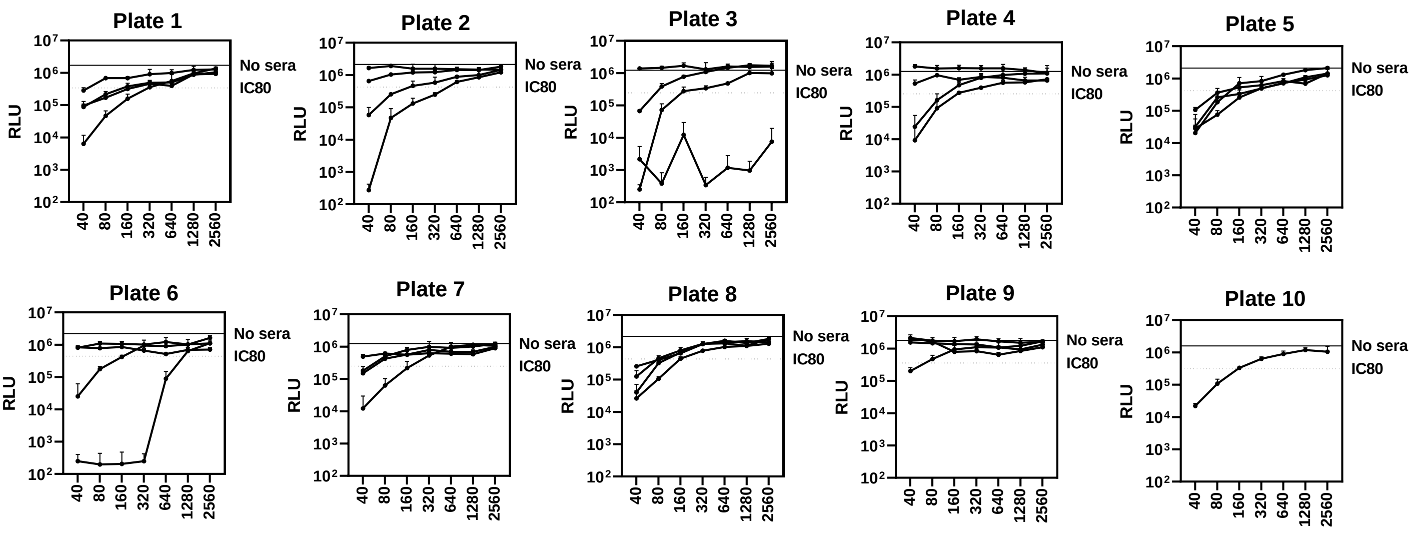

B. Dilution series: 1:10 – 1:80

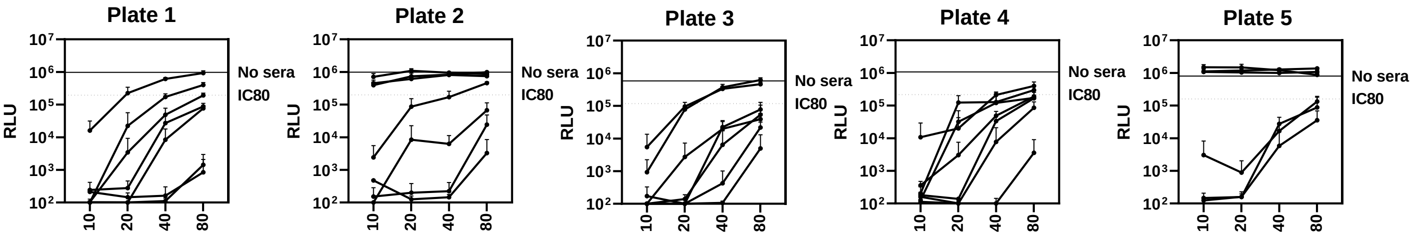

C.

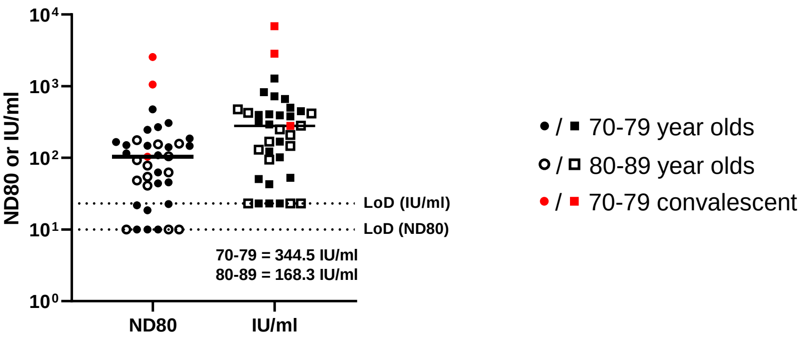

Supplemental Figure 1: Micro virus neutralisation test (mVNT) data examining the neutralising antibody responses generated following BNT162b2 vaccination. Neutralisation titres in **Fig.1-3** were calculated using pseudotypes bearing various SARS-CoV-2 Spikes and sera from a cohort of BNT162b2 vaccinated individuals (n=37 biologically independent samples), recruited as part of the UK CONSENSUS trial, aged 70-89. Titres are expressed as serum fold-dilution required to achieve 80% virus neutralisation, with the titre (ND80) calculated by *xy* interpolation from the mVNT data series (dilution, *x* versus luciferase activity, RLU, *y*). Provided in **(A)** for illustrative and representative purposes are the graphed data from mVNTs (n=37) of D614 (Lineage B) pseudotypes using the sera collected from this cohort at 3 weeks post 2nd dose, diluted 1:40 to 1:2560 (**Fig.1A-B**). **(B)** Results from repeated mVNTs with the same sera panel (n=37), using instead a dilution series of 1:10 to 1:80 to detect lower levels of neutralising antibodies. Subsequent dilution series were adjusted to extend the dilution series. Each graph represents data from a separate 96-well plate used for mVNT, with the plate specific 'no sera' and 'ND80' values highlighted. Mean and standard deviation are plotted for 3 technical replicates. **(C)** mVNTs for these samples were run at the same time as the WHO's International Standard (IS) for SARS-CoV-2 serological assays (NIBSC code: 20/136), to calculate IU/ml values across the experiment. Both the original ND80 titre (round symbol) and IU/ml (square symbol) values are plotted (ages 70-79, n=24, solid symbols; 80-89, n=13, open symbols). Symbols in red represents samples taken from individuals who tested positive for SARS-CoV-2 Nucleoprotein by ELISA, indicative of previous infection. Median is indicated with a solid horizontal line. The relative detection limit of each assay is indicated with a horizontal dotted line.

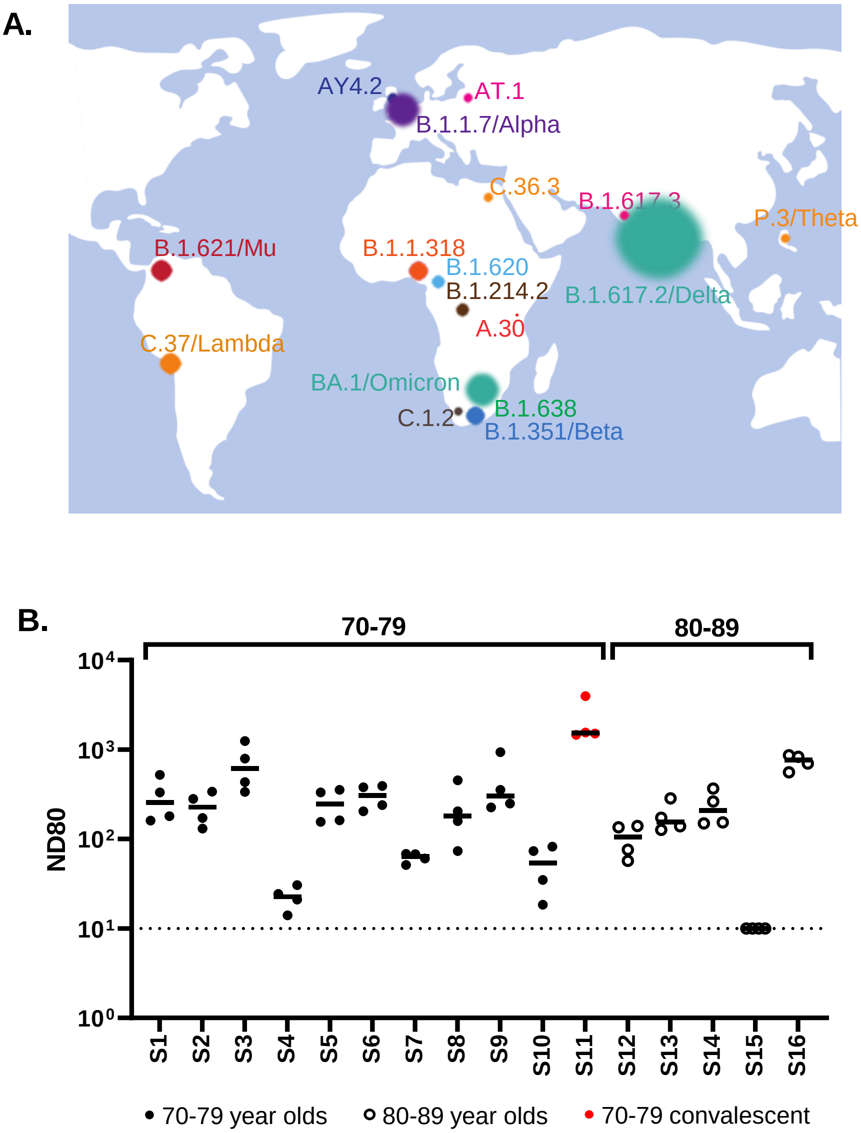

Supplemental Figure 2: Antigenic assessment of SARS-CoV-2 variants. A. World map illustrating where SARS-CoV-2 variants were first detected. **B.** Repeat investigation of SARS-CoV-2 B.1 neutralisation indicates good concordance between assays. Neutralisation titres against B.1 reported in Fig.1 and Fig.2 were repeated across four experiments with the same sub-selection (n=16 biologically independent samples) of the BNT162b2-vaccinated cohort (ages 70-79, solid circles; 80-89, open circles). Plotting of these ND80 titres indicates high concordance, and robust repeatability for the mVNT assay. Symbols in red represents samples taken from individuals who tested positive for SARS-CoV-2 Nucleoprotein by ELISA, indicative of previous infection. The detection limit of the assay is defined as a titre of 10 and is indicated with a dotted line.
