# Supplementary material for: Neutralizing antibody activity against 21 SARS-CoV-2 variants in older adults vaccinated with BNT162b2
Source: Nat Microbiol. 2022 Jul 14;7(8):1180–8. doi: 10.1038/s41564-022-01163-3 (PMC9352594; doi:10.1038/s41564-022-01163-3)
Supplement: Supplementary file 2 — Reporting Summary [file 41564_2022_1163_MOESM2_ESM.pdf]

DBPR
NMICROBIOL-21112852B
Corresponding author(s): Dalan Bailey
Last updated by author(s): May 12, 2022

Reporting Summary

Nature Portfolio wishes to improve the reproducibility of the work that we publish. This form provides structure for consistency and transparency in reporting. For further information on Nature Portfolio policies, see our Editorial Policies and the Editorial Policy Checklist.

Statistics

For all statistical analyses, confirm that the following items are present in the figure legend, table legend, main text, or Methods section.

n/a	Confirmed	
☐	☒	The exact sample size (n) for each experimental group/condition, given as a discrete number and unit of measurement
☐	☒	A statement on whether measurements were taken from distinct samples or whether the same sample was measured repeatedly
☐	☒	The statistical test(s) used AND whether they are one- or two-sided<br>*Only common tests should be described solely by name; describe more complex techniques in the Methods section.*
☒	☐	A description of all covariates tested
☐	☒	A description of any assumptions or corrections, such as tests of normality and adjustment for multiple comparisons
☐	☒	A full description of the statistical parameters including central tendency (e.g. means) or other basic estimates (e.g. regression coefficient) AND variation (e.g. standard deviation) or associated estimates of uncertainty (e.g. confidence intervals)
☐	☒	For null hypothesis testing, the test statistic (e.g. F, t, r) with confidence intervals, effect sizes, degrees of freedom and P value noted<br>*Give P values as exact values whenever suitable.*
☒	☐	For Bayesian analysis, information on the choice of priors and Markov chain Monte Carlo settings
☒	☐	For hierarchical and complex designs, identification of the appropriate level for tests and full reporting of outcomes
☐	☒	Estimates of effect sizes (e.g. Cohen's d, Pearson's r), indicating how they were calculated

Our web collection on statistics for biologists contains articles on many of the points above.

Software and code

Policy information about availability of computer code

Data collection	GloMax Discover System v3.2.3;
Data analysis	Excel v2111 (Microsoft 365); GraphPad Prism v9 (GraphPad Software); Racmacs v1.1.12 (R package)

For manuscripts utilizing custom algorithms or software that are central to the research but not yet described in published literature, software must be made available to editors and reviewers. We strongly encourage code deposition in a community repository (e.g. GitHub). See the Nature Portfolio guidelines for submitting code & software for further information.

Data

Policy information about availability of data

All manuscripts must include a data availability statement. This statement should provide the following information, where applicable:

- Accession codes, unique identifiers, or web links for publicly available datasets
- A description of any restrictions on data availability
- For clinical datasets or third party data, please ensure that the statement adheres to our policy

The datasets generated during and/or analysed during the current study are available from the corresponding author on reasonable request. Unique identifiers cannot be provided for the CONSENSUS trial data for individual participants, due to participant confidentiality, as stated in the PHE/UKHSA study approval documentation sections 4.3.5 and 7.1.

Field-specific reporting

Please select the one below that is the best fit for your research. If you are not sure, read the appropriate sections before making your selection.

☒ Life sciences ☐ Behavioural & social sciences ☐ Ecological, evolutionary & environmental sciences

For a reference copy of the document with all sections, see nature.com/documents/nr-reporting-summary-flat.pdf

Life sciences study design

All studies must disclose on these points even when the disclosure is negative.

Sample size	This sample size was not based on a power calculation as all available samples (n=37) were included in the initial section of the study with VOCs. A smaller representative pool of sera from the same cohort (n=16 total) was selected for wider analysis by ranking the neutralisation responses of the whole cohort based on the Wuhan/Beta IC80 ratio, then selecting evenly ranked samples excluding the samples with the highest and lowest ratio.
Data exclusions	Two sera were excluded from cartography mapping as their titres were consistently below the predetermined detection limit.
Replication	A D614G pseudotype control was included in every neutralisation experiment as a reference, replicated a total of 5 times; these independent repeats showed good concordance and robust repeatability between experiments. Experiments for other individual variants were not replicated due to the finite availability of samples, but were run in biological triplicates within each experiment.
Randomization	We tested every available sera from the control arm of the CONSENSUS study in our initial experiments (n=37) so there was no possibility to choose a random selection. For subsequent experiments using smaller pools of sera, we state in the manuscript the randomization process "Using a smaller pool of sera from the same cohort (3-weeks post 2nd dose; n=16 total; 70-79, n=11; 80-89 n=5), selected by ranking the neutralisation ND80 ratio of the lineage B virus to Beta across the whole cohort and picking evenly ranked serum samples"
Blinding	Blinding was not possible or applicable to this study as experiments were performed in an unbiased manner. Researchers were unaware of an expected outcome, so would not be able to influence it.

Reporting for specific materials, systems and methods

We require information from authors about some types of materials, experimental systems and methods used in many studies. Here, indicate whether each material, system or method listed is relevant to your study. If you are not sure if a list item applies to your research, read the appropriate section before selecting a response.

Materials & experimental systems

n/a	Involved in the study	
☐	☒	Antibodies
☐	☒	Eukaryotic cell lines
☒	☐	Palaeontology and archaeology
☒	☐	Animals and other organisms
☐	☒	Human research participants
☒	☐	Clinical data
☒	☐	Dual use research of concern

Methods

n/a	Involved in the study	
☒	☐	ChIP-seq
☒	☐	Flow cytometry
☒	☐	MRI-based neuroimaging

Antibodies

Antibodies used	Anti-human IgG horseradish peroxidase antibody conjugate (AP112P, Sigma-Aldrich, Poole, UK), diluted 1:15,000
Validation	*Describe the validation of each primary antibody for the species and application, noting any validation statements on the manufacturer's website, relevant citations, antibody profiles in online databases, or data provided in the manuscript.*

Eukaryotic cell lines

Policy information about cell lines

Cell line source(s)	HEK293T (ATCC CRL-11268G-1), Vero-E6-TMPRSS2 (King's College London, Stuart JD Neil)
Authentication	Cell lines were not authenticated, although they were purchased from ATCC orginally. The morphology of the cell lines appear as expected.
Mycoplasma contamination	Cell lines tested negative for mycoplasma contamination.

March 2021

Commonly misidentified lines (See ICLAC register)	None used.

Human research participants

Policy information about studies involving human research participants

Population characteristics	We are only able to provide aggregated data for this cohort due to patient confidentiality. We have provided the aggregated information in supplementary datasheet 1. The media age of this cohort was 78 [75-80 IQR], we had a make-up of 54.1% females, 45.9% males. Information about the dates of COVID-19 vaccine are provided in supplementary datasheet 1.
Recruitment	The CONSENSUS study aimed to recruit healthy participants through London Primary Care Networks (PCN) in 10-year age bands prioritising those over 60 years old. Sera for this study were from some of the first vaccinees in the UK, where participants had received 2 dose doses of Pfizer-BioNTech vaccine 3 weeks apart (control arm).<br><br>Participants were recruited on a voluntary basis when attending a vaccination clinic vaccination. We do not expect the recruitment to introduce a bias in the analyses described int the paper.
Ethics oversight	The protocol was approved by Public Health England Research Ethics Governance Group (reference NR0253; 18/01/21).<br><br>Participants who were unable to provide informed written consent were excluded from the recruitment process.

Note that full information on the approval of the study protocol must also be provided in the manuscript.
